# Carbapenem-Resistant *Acinetobacter baumannii* Bloodstream Infection in a Ghanaian Patient with Unilateral Diaphragmatic Eventration and HIV Type 1 Infection

**DOI:** 10.1155/2023/9930291

**Published:** 2023-10-12

**Authors:** Yvonne Ayerki Nartey, Augustine Boakye Donkor, Ampem Darko Jnr Siaw, Oluwayemisi Esther Ekor, Bashiru Babatunde Jimah

**Affiliations:** ^1^Department of Medicine, Cape Coast Teaching Hospital, Cape Coast, Ghana; ^2^Department of Internal Medicine, School of Medical Sciences, University of Cape Coast, Cape Coast, Ghana; ^3^Department of Clinical Microbiology, Cape Coast Teaching Hospital, Cape Coast, Ghana; ^4^Department of Anaesthesia and Pain Management, School of Medical Sciences, University of Cape Coast, Cape Coast, Ghana; ^5^Department of Medical Imaging, School of Medical Sciences, University of Cape Coast, Cape Coast, Ghana

## Abstract

Carbapenem-resistant *Acinetobacter baumannii* infection is a critically prioritized pathogen by the World Health Organization and a cause for growing concern due to increased mortality among hospitalised patients. Phrenic nerve palsy is a rare complication of herpes zoster infection of the C3, C4, and C5 nerve roots. We present a case of bloodstream carbapenem-resistant *A. baumannii* infection in a Ghanaian patient with HIV type 1 infection and multiple risk factors, including unilateral diaphragmatic eventration with compression atelectasis likely secondary to phrenic nerve palsy due to herpes zoster infection, consequently leading to recurrent hospital and ICU admission. In this case, we emphasize the need for clinicians in LMICs to be aware of CRAB, in order to advocate for the availability of evidence-based medicines in resource-limited settings for appropriate treatment. In addition, we illustrate the importance of a high index of suspicion for infection with carbapenem-resistant organisms such as *A. baumannii* and highlight a rare and severe complication of herpes zoster infection in the form of phrenic nerve palsy and consequent diaphragmatic eventration.

## 1. Introduction

Carbapenems are bactericidal antibiotics which act by binding irreversibly to the active site of the penicillin-binding proteins (PBPs) such as PBP1a, 1b, 2, and 3 [1, 2]. Carbapenems are the antibacterial agents of choice for severe and complicated community-acquired infections [3]. They are also used to treat significant hospital-acquired infections [4]. Carbapenems are well tolerated and have a high safety profile [5]. Generally, they are considered as last-resort antibiotics and prescribed for critically ill patients, most often with infections caused by multidrug-resistant (MDR) pathogens such as *Acinetobacter baumannii* [6].

*A*. *baumannii* is a Gram-negative, aerobic nonfastidious bacterium and exhibits the following biochemical reactions: indole-negative, nonmotile (with some strains exhibiting twitching and surface motility), catalase-positive, oxidase-negative, and citrate-positive [7]. *A*. *baumannii* mostly inhabits in hospital microenvironments such as adult and paediatric ICUs, burn units, neurosurgical and general surgical wards, and medical and oncology units [8]. They are commonly isolated from skin and soft tissue infections, respiratory tract infections, bloodstream infections, central nervous system infections, surgical site infections, and catheter-associated urinary tract infections as well as from ventilator-associated pneumonia (VAP) [9, 10]. Risk factors for the acquisition of MDR *A. baumannii* strain included prolonged mechanical ventilation, longer ICU or hospital stay, vulnerability to infected patients, increased seriousness of disease, and administration of broad-spectrum antibiotics, specifically third-generation cephalosporins, fluoroquinolones, and carbapenems [10–13].

Diaphragmatic eventration is described as elevation and malposition of the diaphragm which may be unilateral or bilateral and may result from congenital or acquired causes [14]. Acquired causes include trauma, surgical procedures of the thorax, and phrenic nerve dysfunction due to compression, inflammation (e.g., acute inflammatory demyelinating polyneuropathy, and multiple sclerosis), and rarely infections such as herpes zoster involving the C3, C4, and C5 spinal nerves, which innervate the diaphragm [15, 16].

Here, we present a case of an HIV type 1-infected patient with herpes zoster-associated phrenic nerve palsy leading to unilateral diaphragmatic eventration and compression atelectasis, which consequently led to recurrent hospital and ICU admission, predisposing her to bloodstream carbapenem-resistant *Acinetobacter baumannii* infection.

## 2. Case Presentation

A 68-year-old woman presented to the Cape Coast Teaching Hospital with a 5-day history of nonproductive cough, difficulty breathing, and chest pain. There was no associated fever; however, she experienced palpitations, dyspnea on exertion, and orthopnea. The patient had previously been admitted to a primary care facility one week prior to presentation and managed for pneumonia and congestive cardiac failure secondary to hypertension. She had also been managed at a tertiary referral center for a similar presentation 2 months prior to her admission at our hospital. Her past medical history was significant for systemic arterial hypertension for 20 years, for which she was compliant on antihypertensive medication.

On examination, an obese woman was seen in acute respiratory distress, with a herpetic rash on the left side of her neck and anterior chest wall in the C3, C4, and C5 dermatome. She was afebrile, normotensive, and saturating at 85–88% in room air. She was tachypneic, with a respiratory rate of 28 cycles per minute. On respiratory examination, the patient was found to have reduced breath sound intensity, bronchial breath sounds, and a stony dull percussion note in the left middle and lower lung zones. She had a diffuse apex beat and no cardiac murmurs. A bedside point-of-care ultrasound scan demonstrated a moderate left pleural effusion, and an ultrasound-guided tube thoracostomy was performed in the emergency room, which drained serosanguinous fluid. The patient was subsequently transferred to the intensive care unit and placed on bilevel positive airway pressure (BiPAP). She was commenced on IV ceftriaxone and oral azithromycin as per hospital ICU protocol.

**Figure 1:**
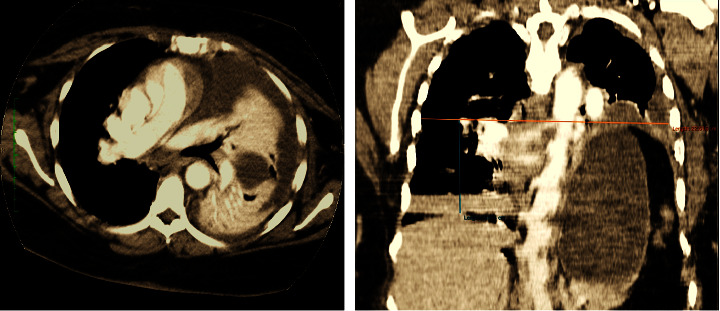
Axial and coronal reformatted CT scan images of the chest, mediastinal window. The images show dense consolidation with air bronchograms in the left lower lobe consistent with pneumonia. There is associated significant fluid in the left pleural space, in keeping with parapneumonic effusion. The coronal image shows ∼7.8 cm elevation of the left hemidiaphragm relative to the right hemidiaphragm, resulting in severe left lung volume loss and mediastinal shift to the right.

Initial investigations showed an elevated white blood cell count of 13.68 × 10^9^/L with neutrophilia and normocytic normochromic anaemia. Cardiac troponin, renal, and liver function tests were unremarkable; however, the D-dimer was elevated with a value of 8.13 *μ*g/mL. HIV First Response® was positive for HIV type 1 infection; however, the patient refused follow-up testing and counseling. COVID-19 antigen testing, sputum for acid-fast bacilli, and GeneXpert® were all negative. The erythrocyte sedimentation rate was 31 mm/hour, and the C-reactive protein level was 148.7 mg/L. Her ECG showed sinus tachycardia, and an echocardiogram demonstrated good biventricular systolic function, diastolic dysfunction type 1, mild pericardial effusion, and an ejection fraction of >50%. There was no evidence of pulmonary hypertension. A chest and abdominal CT scan with pulmonary angiogram revealed extensive left lower lobar pneumonia with parapneumonic effusion and severe left diaphragmatic eventration (severe elevation of the left hemidiaphragm) with compressive atelectasis of the left lower lobe and severe volume loss of the left hemithorax (Figure 1). There was no evidence of pulmonary embolism. Initial aerobic and anaerobic cultures of blood, sputum, and pleural aspirate yielded no bacterial growth. Surgical consult was sought for her CT findings, and supportive management was advised by the surgeons.

The patient failed to improve clinically on IV ceftriaxone with persistent elevation of inflammatory markers and white blood cell indices, and antibiotics were switched to IV meropenem as per hospital ICU protocol, with repeat cultures taken. However, the patient failed to improve clinically on IV meropenem and developed a fever on day 10 of admission. Repeat blood cultures isolated the Gram-negative bacilli *Acinetobacter baumannii* (*anitratus*), which demonstrated multidrug resistance, including third- and fourth-generation cephalosporins and carbapenems. The antibiogram is shown in Table 1. The patient was commenced on IV amikacin for 7 days and her temperatures settled after 2 days of its initiation, with subsequent reduction in white cell indices and inflammatory markers. The patient was discharged 23 days after admission for follow-up care with the infectious disease specialist and the cardiothoracic surgeons on account of the severe left diaphragmatic eventration. She returned for review one week after discharge with no respiratory symptoms.

## 3. Discussion

In this patient, the history of recurrent hospital admissions, HIV infection, prior betalactam therapy, ICU admission, noninvasive ventilation, invasive procedures (central venous catheter and tube thoracostomy), and her anatomic pathology (diaphragmatic eventration) are probable factors which made her susceptible to infection with carbapenem-resistant *Acinetobacter baumannii* (CRAB). It is possible that her recurrent hospital presentations with orthopnea and dyspnea on exertion were related to the diaphragmatic eventration secondary to phrenic nerve palsy, which may have been previously missed or determined to be due to congestive cardiac failure. Phrenic nerve palsy as a complication of herpes zoster affecting the cervical nerve root distribution occurs because of reactivation of the virus in persons with immunocompromised states such as old age and HIV infection [16]. The patient did not present with any previous chest X-rays but reported she had never been told of an anatomic pathology by her previous doctors. Respiratory and GI symptoms are a common presentation of phrenic nerve palsy and diaphragmatic eventration, and the pathology is often complicated by recurrent lung infections and lung atelectasis [14], as was found in this patient. Her recurrent treatment and exposure to healthcare settings are therefore likely to have made her susceptible to *A. baumannii* infection.

*A. baumannii* is part of the ESKAPE group of bacteria which are often the cause of healthcare-associated infections and are responsible for significant mortality in hospitalised patients. Failure of our patient to respond to cephalosporin and carbapenem therapy increased the likelihood and suspicion that this patient was infected with a multidrug-resistant organism.

There is growing evidence of carbapenem-resistant *Acinetobacter baumannii* infection from clinical isolates from the surgical site and urinary specimens in Ghana [17, 18]; however, few studies or case reports have demonstrated bacteraemia associated with carbapenem-resistant *Acinetobacter baumannii*. A case of a Ghanaian infant with malaria and multidrug-resistant *A. baumannii* bloodstream infection was previously reported, but the isolate was not tested against carbapenems [19]. In investigating the antibiotic resistance profile and genotypic characteristics of Acinetobacter isolates from Ghana and to characterize carbapenemase producers using whole-genome sequencing (WGS), Ayibieke and his colleagues showed that *A. baumannii* isolates of diverse and unique genotypes, including OXA-carbapenemase producers, were circulating in Ghana [17].

In recent times, there has been over 40% increase in CRAB infections in most hospitals, especially in ICU settings [20]. The mechanisms that render *A. baumannii* resistant to carbapenem include alterations in penicillin-binding proteins [7, 21], loss of outer membrane porins [22–24], overexpression of efflux pumps [22, 23, 25], and synthesis of carbapenemases (carbapenem-hydrolysing *β*-lactamases) [23, 26].

To overcome carbapenem resistance, a multidimensional program in hospitals can be implemented to decrease the occurrence and chance of outbreaks such as improved infection control programs and practices, surveillance cultures, and a dedicated antimicrobial stewardship program [27]. Therapeutic antibiotics for treating carbapenem-resistant *A. baumannii* infections are aminoglycosides such as amikacin and cell membrane inhibitors such as polymyxin or colistin, *β*-lactamase inhibitors, and tetracyclines such as tigecycline and combination of trimethoprim (TMP)-sulfamethoxazole (SMX) [28–30]. Although amikacin is a good choice of therapy and relatively affordable in LMICs, intravenous administration is associated with dose-related toxicity, with risks of ototoxicity and nephrotoxicity possible among those treated [31]. The cure rate of amikacin against CRAB has been reported as 46.9%, with antibiotics such as tigecycline and colistin demonstrating superior rates [32]. Agents such as polymixin, which have well-documented in vitro activity, are limited by treatment failures, suboptimal drug profiles, re-emergence of resistance, and/or associated toxic effects [33]. Furthermore, colistin has been shown to have a more favorable outcome in CRAB treatment depending on the dosing regimen, treatment duration, and additional antibiotics chosen for combined therapy. Katip and his colleagues demonstrated in a retrospective cohort analysis of 383 patients that the administration of colistin methanesulfonate through a loading dose rather than a nonloading dose regimen led to enhanced bacterial killing [34]. In addition, a longer duration of therapy (>14 days) was associated with lower 30-day mortality [35]. Several combination therapy regimens have been proposed with colistin as the mainstay including colistin-meropenem and colistin-imipenem [36–38], as well as ampicillin-sulbactam and sulbactam-durlobactam [38]. Comparing the synergistic therapies with imipenem or meropenem, the combination of loading dose colistin with meropenem has been shown to yield favorable outcomes in the treatment of CRAB [36]. Evidence shows reduced 7-day and 30-day mortality [32, 35], superior clinical and microbiological outcomes, and indifferent nephrotoxicity profiles with colistin-meropenem combination therapy compared to using colistin alone [35]. Interestingly, the combination of colistin with vancomycin has not demonstrated improved outcomes when compared to monotherapy [39].

Evidence suggests that CRAB therapy must be individualized based on host factors, local susceptibility patterns, and pharmacologic principles [33]. In our patient, the choice to undertake amikacin monotherapy was based on lack of access to tigecycline or colistin in our resource-limited setting and demonstrates the need for awareness of CRAB infections which require evidence-based effective combination therapy. Appropriate therapy is important due to the high mortality associated with the condition. Ninety-day mortality has been reported at 30.3% in the USA and 61.8% in Turkey [40, 41], with higher mortality associated with the presence of comorbidities and inappropriate use of empirical antibiotics [42]. Given the high mortality even in well-resourced settings, it is imperative that clinicians in resource-limited settings recognize CRAB infection in a timely manner with relevant considerations for evidence-based therapy, in order to improve survival.

## 4. Conclusions

In this case report, we describe for the first time in Ghana a case of CRAB bacteraemia in an immunocompromised adult patient and emphasize the need for clinicians to be aware of CRAB, in order to advocate for the availability of evidence-based medicines in resource-limited settings for appropriate treatment. Furthermore, we illustrate the need for clinicians to have a high index of suspicion for infection with carbapenem-resistant organisms such as *A. baumannii* in patients with multiple risk factors and who fail to respond to initial antibiotic therapy. We additionally highlight a rare and severe complication of herpes zoster infection in the form of phrenic nerve palsy and consequent diaphragmatic eventration.

## Figures and Tables

**Table 1:** Antibiogram of blood culture isolate *A. baumannii*.

Antibiotic	Interpretation
Cephalosporins	
Ceftazidime	R
Cefepime	R
Beta-lactamase inhibitors	
Piperacillin/tazobactam	R
Carbapenems	
Imipenem	R
Meropenem	R
Aminoglycosides	
Amikacin	S
Gentamicin/netilmicin	R
Tobramycin	R
Quinolones	
Ciprofloxacin	R
Others	
Tigecycline	S

*S* = susceptible; *R* = resistant.

## Data Availability

Data underlying the case report are available as part of the article and no additional patient information can be shared due to patient privacy.
